# Evaluation of the diagnostic efficacy of core biomarkers in cerebrospinal fluid for Alzheimer’s disease: a systematic review and meta-analysis

**DOI:** 10.3389/fneur.2025.1667402

**Published:** 2025-09-30

**Authors:** Baohua Deng, Yangtai Zheng, Yiyang Gao, Yafan Zhuang, Li Ma, Lihui Cao

**Affiliations:** ^1^Biomedical Innovation and Entrepreneurship Laboratory, Jinan University, Guangzhou, China; ^2^School of Medicine, Jinan University, Guangzhou, China; ^3^College of Life Science and Technology, Jinan University, Guangzhou, China; ^4^Department of Pharmacy, Guangdong Provincial Key Laboratory of Major Obstetric Diseases, Guangdong Provincial Clinical Research Center for Obstetrics and Gynecology, The Third Affiliated Hospital, Guangzhou Medical University, Guangzhou, China

**Keywords:** Bayesian meta-analysis, Alzheimer’s disease, core cerebrospinal fluid biomarkers, *β*-Amyloid protein, tau protein, diagnostic performance evaluation

## Abstract

**Objective:**

Core cerebrospinal fluid (CSF) biomarkers serve as pivotal diagnostic indicators for Alzheimer’s disease, yet their diagnostic efficacy varies substantially across different markers. This study employs a Bayesian meta-analytic approach to comprehensively evaluate the diagnostic accuracy of individual core CSF biomarkers for Alzheimer’s disease (AD).

**Methods:**

A comprehensive literature search was conducted in Web of Science, PubMed, and other databases for English-language studies published between January 2013 and April 2025. Following predefined inclusion/exclusion criteria, eligible studies were selected for methodological quality assessment using standardized tools. Data extraction was subsequently performed, and statistical analyses were conducted using Meta-DiSc 1.4, Stata 15.1, and R 4.3.2 software packages.

**Results:**

This meta-analysis systematically evaluated the diagnostic value of eight core CSF biomarkers for AD, incorporating 23 eligible studies (2,187 AD cases and 2,019 non-AD). Key findings revealed: (1) Among individual biomarkers, p-tau217 demonstrated superior diagnostic performance, with sensitivity of 0.95 (95% CI: 0.92–0.97), specificity of 0.94 (95% CI: 0.88–0.98), area under the curve (AUC) of 0.99 (95% CI: 0.97–1.00), and an exceptionally high diagnostic odds ratio (DOR) of 395.28 (95% CI: 92.17–1,305.79), all parameters being significantly better than other biomarkers (*p* < 0.001). Both p-tau231 (AUC = 0.97) and p-tau181 (AUC = 0.90) also exhibited commendable diagnostic accuracy. (2) For biomarker ratios, the Aβ42/p-tau181 ratio showed optimal overall diagnostic efficacy, with sensitivity of 0.90 (95% CI: 0.86–0.94) and AUC of 0.93 (95% CI: 0.90–0.96), significantly outperforming other ratio combinations (*p* < 0.05).

**Conclusion:**

This study demonstrates that among core CSF biomarkers for AD, p-tau217 exhibits the most outstanding diagnostic performance as a standalone biomarker, while the Aβ42/p-tau181 ratio shows superior diagnostic efficacy among biomarker combinations. Based on current evidence-based medical data, we recommend the combined use of p-tau217 and Aβ42/p-tau181 ratio as first-line core CSF biomarker panel for AD diagnosis, providing reliable laboratory evidence for early screening and differential diagnosis of AD in clinical practice.

## 1 Introduction

Alzheimer’s disease (AD) is a progressively developing neurodegenerative disorder (1, 2), for 60 to 80% of all cases of cognitive impairment (3). With the continued progression of global population aging, it is projected that the number of individuals diagnosed with dementia worldwide will increase significantly, reaching an estimated 152 million by the year 2050 (4–6). Although the currently available drugs for treating AD exhibit some efficacy in delaying disease progression, their capacity to provide a complete cure remains limited (7). Consequently, researchers are placing greater emphasis on exploring innovative strategies for the prevention and diagnosis of AD by focusing on risk factors and early diagnostic methods.

The pathological features of AD encompass the abnormal accumulation of extracellular *β*-amyloid protein (Aβ), the formation of intracellular neurofibrillary tangles composed of phosphorylated tau protein (p-tau181), and the resultant neuronal loss (8, 9). In recent years, the detection of biomarkers in CSF, such as amyloid PET, the ratio of Aβ42 to Aβ40 (Aβ42/Aβ40), the ratio of Aβ42 to p-tau181 (Aβ42/p-tau181), and the ratio of Aβ42 to total tau (Aβ42/t-tau), has played a key role in the initial diagnosis of AD (10). Furthermore, other biomarkers, including phosphorylated tau (p-tau), total tau (t-tau), RBKS, PDGFC, NUCB2, TNFSF14, CASP3, and SQSTM1/p62, are also considered potential supplements or substitutes for biomarkers in the diagnostic composite reference criteria (10–12). Although several studies have reported the potential of these biomarkers in predicting the progression of AD, enabling the identification of patients at an early stage, monitoring the disease process, and evaluating drug efficacy, there is currently a lack of systematic evaluation and diagnostic meta-analysis comparing different biomarkers suitable for the differential diagnosis of AD. Previous reviews have either focused on a single biomarker or neglected to conduct comparative analyses of cerebrospinal fluid samples under real-life conditions or in professional care settings, thereby failing to evaluate their diagnostic accuracy for AD (13, 14).

This study employed the Bayesian meta-analysis method to systematically evaluate the diagnostic accuracy of core CSF for AD. This approach effectively accommodates the challenges posed by small sample sizes and complex data by allowing for flexible prior distribution settings, thereby significantly enhancing the model’s estimation accuracy. This research not only provides an in-depth analysis of the diagnostic mechanisms of biomarkers but also accurately identifies the potential factors influencing their efficacy. Furthermore, it offers a theoretical basis and practical value for enhancing the diagnostic accuracy of AD and guiding clinical practice.

## 2 Methods

### 2.1 Search strategy

This investigation was prospectively registered on the PROSPERO international prospective register of systematic reviews (Registration No. CRD420251021602), with strict adherence to the Preferred Reporting Items for Systematic Reviews and Meta-Analyses (PRISMA) guidelines. A comprehensive electronic literature search was conducted in the Web of Science Core Collection and PubMed/MEDLINE databases, encompassing studies published between January 2013 and April 2025. The search strategy employed a structured Boolean query combining Medical Subject Headings (MeSH) terms and free-text keywords with the following syntax: (“Biomarker” OR “Diagnostic Test” OR “Alzheimer’s disease” [MeSH] OR “Cerebrospinal Fluid” [MeSH] OR “Aβ42” OR “p-tau181” OR “p-tau217” OR “p-tau231” OR “p-tau205” OR “t-tau”).

### 2.2 Inclusion and exclusion criteria

Studies were selected for inclusion in this meta-analysis based on the following rigorous criteria: (1) original diagnostic accuracy studies evaluating the performance of core CSF biomarkers in differentiating AD from other neurological disorders in clinical settings; (2) application of validated diagnostic criteria for both AD and non-AD control groups including mild cognitive impairment (MCI), Lewy body dementia, Parkinson’s disease dementia, and other neurodegenerative conditions; (3) comprehensive reporting of sample characteristics, analytical methods, reference standards, and sufficient data to construct complete diagnostic contingency tables, with clearly defined cutoff values derived either from established clinical thresholds or training datasets; (4) no restrictions on publication date, language, patient demographics, or reference standards to ensure global representation of evidence; and (5) primary focus on core CSF AD pathological markers including amyloid-*β* isoforms (e.g., Aβ42), tau protein (e.g., t-tau and p-tau species), and their clinically validated ratios (e.g., Aβ42/Aβ40, Aβ42/p-tau181).

Studies were systematically excluded from this meta-analysis based on the following predefined criteria: (1) investigations employing exclusively peripheral blood biomarkers or neuroimaging modalities without concurrent CSF biomarker analysis; (2) studies utilizing healthy controls as the sole comparator group rather than defined neurological disorders; (3) The data is incomplete, making it impossible to directly extract or compute the necessary information to construct the diagnostic contingency table; (4) preclinical research limited to animal models; and (5) non-diagnostic studies including purely mechanistic, pathological, or therapeutic investigations.

### 2.3 Data extraction

First, remove duplicate references using reference management software. Subsequently, two independent reviewers screen the remaining references by evaluating their abstracts and full texts. In cases of disagreement, a third reviewer is consulted to resolve discrepancies. The extracted data comprise the author’s name, publication year, sample size, as well as the sensitivity, specificity, AUC values, and detection methods for Aβ42, t-tau, p-tau181, p-tau217, p-tau231, Aβ42/Aβ40, Aβ42/t-tau, and Aβ42/p-tau181.

### 2.4 Assessment of literature quality

QUADAS-2 encompasses four key areas: patient selection, index test, reference standard, and flow and timing. Each area contains specific signaling problems that aid in identifying potential risks of bias and applicability issues. The response options for signaling problems in each area are typically “Yes,” “No,” or “Unclear.” Based on the responses to these questions, the risk of bias and applicability can be categorized as “low,” “high,” or “unclear,” respectively.

### 2.5 Data analysis

All statistical analyses were performed using Meta-Disc 1.4, Stata 15.1, and R 4.3.2. A contingency table was employed to derive the values of True Positive, False Positive, False Negative, and True Negative for each study included in the analysis, facilitating the evaluation of the diagnostic tests’ accuracy. Heterogeneity tests were conducted to assess the variability among studies, utilizing the I^2^ statistic for this purpose. An I^2^ < 50% indicated low heterogeneity, warranting the use of a fixed-effect model. Conversely, an I^2^ > 50% suggested high heterogeneity, necessitating the adoption of random effects models. The analysis encompassed sensitivity, specificity, positive likelihood ratio (LR+), negative likelihood ratio (LR−), diagnostic odds ratio (DOR), and the area under the receiver operating characteristic curve (AUC).

## 3 Results

### 3.1 Study search results

**Figure 1:**
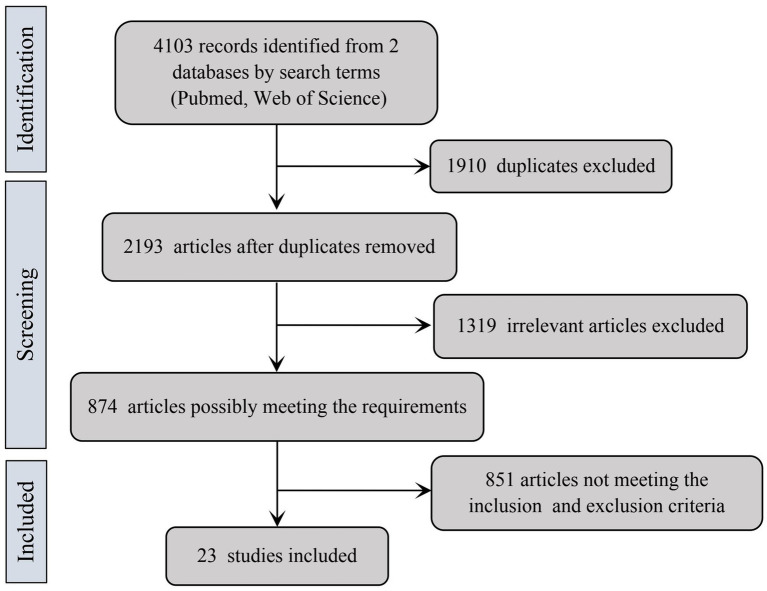
The flowchart of study selection.

Our systematic search identified 4,103 potentially relevant publications from electronic databases. After removing 1,910 duplicate records, we excluded 1,319 articles through abstract screening based on predefined inclusion/exclusion criteria. Of the remaining 874 full-text articles assessed for eligibility, 23 studies met all selection criteria and were included in the final analysis (see Figure 1). The analysis encompassed eight distinct core CSF biomarkers, with pooled data from 2,187 AD patients and 2,019 non-AD dementia controls. Detailed characteristics of included studies are presented in Table 1.

**Table 1:** General characteristics of included publications.

Study	Case sample size		CSF biomarker	Sen	Spc	TP	FP	FN	TN	Cut off	AUC	Technology
	AD	non-AD										
Slaets et al. (25)	80	75	Aβ42	0.81	0.59	65	31	15	44	517	0.747	ELISA
			Aβ42/Aβ40	0.81	0.6	65	30	15	45	0.057	0.749	
Dumurgier et al. (26)	160	207	Aβ42	0.78	0.79	125	43	35	164	752	0.81	ELISA
			t-tau	0.84	0.81	134	39	26	168	330	0.87	
			p-tau181	0.84	0.81	134	39	26	168	62	0.9	
			Aβ42/Aβ40	0.73	0.78	117	45	43	162	0.055	0.81	
Struyfs et al. (27)	100	50	Aβ42	0.959	0.4	96	30	4	20	694	0.686	ELISA
			t-tau	0.878	0.64	88	18	12	32	335.5	0.819	
			p-tau181	0.918	0.66	92	17	8	33	50	0.82	
			Aβ42/Aβ40	0.939	0.5	94	25	6	25	0.1215	0.782	
			Aβ42/t-tau	0.878	0.76	88	12	12	38	1.42	0.842	
			Aβ42/p-tau181	0.878	0.72	88	14	12	36	9.44	0.84	
Bousiges et al. (28)	9	13	Aβ42/Aβ40	0.923	0.889	8	1	1	12	0.0799	0.86	ELISA
Constantinides et al. (29)	98	144	Aβ42	0.95	0.669	93	48	5	96	430.7	0.831	ELISA
			t-tau	1	0.845	98	22	0	122	397	0.948	
			p-tau181	0.95	0.873	93	18	5	126	74.8	0.96	
			Aβ42/Aβ40	0.95	0.857	93	21	5	123	0.078	0.939	
			Aβ42/t-tau	1	0.901	98	15	0	129	1.03	0.975	
			Aβ42/p-tau181	0.983	0.84	96	23	2	121	0.155	0.952	
Ortner et al. (30)	86	44	Aβ42	0.855	0.75	74	11	12	33	825	0.812	CLIA
			t-tau	0.494	0.864	43	6	43	38	323	0.706	
			p-tau181	0.843	0.705	73	13	13	31	21	0.793	
			Aβ42/Aβ40	0.867	0.773	75	10	11	34	0.055	0.829	
			Aβ42/t-tau	0.892	0.75	77	11	9	33	0.27	0.822	
			Aβ42/p-tau181	0.892	0.773	77	10	9	34	0.022	0.841	
Oudart et al. (31)	140	240	Aβ42	0.857	0.546	120	109	20	131	706	0.738	ELISA
			t-tau	0.814	0.725	114	66	26	174	355	0.822	
			p-tau181	0.829	0.733	116	64	24	176	57	0.831	
			Aβ42/Aβ40	0.871	0.688	122	75	18	165	0.059	0.823	
Baldeiras et al. (32)	107	107	Aβ42	0.7	0.82	75	19	32	88	538	0.791	ELISA
			t-tau	0.61	0.82	65	19	42	88	300	0.756	
			Aβ42/Aβ40	0.59	0.87	63	14	44	93	5.4	0.778	
			Aβ42/t-tau	0.79	0.84	84	17	23	90	0.77	0.864	
Mazzeo et al. (33)	101	44	Aβ42	0.868	0.486	88	23	13	21	776.34	0.567	CLIA
			t-tau	0.915	0.829	92	8	9	36	509.09	0.941	
			Aβ42/Aβ40	0.882	0.868	89	6	12	38	0.068	0.943	
Keshavan et al. (34)	13	50	Aβ42	1	0.74	13	13	0	37	1,423	0.891	CLIA
			t-tau	0.54	0.82	7	9	6	41	443	0.665	
			p-tau181	1	0.66	13	17	0	33	49	0.879	
			Aβ42/Aβ40	1	0.94	13	3	0	47	0.11	0.966	
			Aβ42/t-tau	0.92	0.90	12	5	1	45	3.167	0.955	
			Aβ42/p-tau181	1	0.94	13	3	0	47	25.25	0.966	
Dorey et al. (35)	281	244	Aβ42/Aβ40	0.762	0.582	214	102	67	142	0.06	0.7	ELISA
Frölich et al. (36)	28	87	Aβ42	0.74	0.64	21	31	7	56	749	0.68	ELISA
			t-tau	0.62	0.86	17	12	11	75	411	0.77	
			Aβ42/Aβ40	0.59	0.75	17	22	11	65	0.08	0.66	
Abildgaard et al. (37)	123	89	Aβ42	0.6	0.92	74	7	49	82	943	0.74	CLIA
			t-tau	0.67	0.87	82	12	41	77	279	0.81	
			p-tau181	0.75	0.88	92	11	31	78	24	0.87	
			Aβ42/t-tau	0.86	0.84	106	14	17	75	0.34	0.9	
			Aβ42/p-tau181	0.84	0.89	103	10	20	79	0.033	0.92	
Struyfs et al. (38)	140	77	Aβ42	0.793	0.532	111	36	29	41	500.27	0.677	ELISA
			t-tau	0.621	0.636	87	28	53	49	472.35	0.592	
			p-tau181	0.779	0.61	109	29	31	48	50.35	0.72	
			Aβ42/t-tau	0.75	0.571	105	33	35	44	1.08	0.678	
			Aβ42/p-tau181	0.829	0.597	116	31	24	46	9.11	0.77	
Chiasserini et al. (39)	48	40	Aβ42	0.79	0.6	38	16	10	24	537	0.6432	ELISA
			t-tau	0.79	0.83	38	7	10	33	461.4	0.8524	
			p-tau181	0.9	0.78	43	9	5	31	69.2	0.8914	
Baiardi et al. (40)	97	168	p-tau181	0.918	0.905	89	16	8	152	65.5	0.954	CLIA
Vergallo et al. (41)	45	20	Aβ42/p-tau181	0.9	0.87	41	3	4	17	4.244	0.924	ELISA
Forlenza et al. (42)	41	35	Aβ42	0.83	0.54	34	16	7	19	416	non	FD
			t-tau	0.78	0.57	32	14	9	21	76.7	non	
			p-tau181	0.8	0.68	33	11	8	24	36.1	non	
			Aβ42/p-tau181	0.83	0.64	35	10	6	25	0.0867	0.8	
Perani et al. (43)	47	14	Aβ42	0.85	0.43	40	8	7	6	515	0.64	ELISA
			t-tau	0.38	0.86	18	2	29	12	350	0.67	
			p-tau181	0.7	0.64	33	5	14	9	52.5	0.62	
			Aβ42/t-tau	0.79	0.68	37	4	10	10	0.6769	0.81	
Leuzy et al. (44)	119	92	p-tau217	0.9386	0.9667	112	3	7	89	190.87	0.98	CLIA
			p-tau231	0.9298	0.9111	111	8	8	84	18.76	0.96	ELISA
Ashton et al. (13)	127	70	p-tau217	0.96	0.951	122	3	5	67	6.15	non	ELISA
			p-tau231	0.882	0.9	112	7	15	63	100.4	non	
Wojdała et al. (45)	35	39	p-tau231	1	0.943	33	2	2	37	20.8	0.98	Simoa
Pilotto et al. (46)	162	70	p-tau217	0.938	0.887	152	8	10	62	0.518	0.955	Simoa

Sen, Sensitivity; Spc, Specificity; FD, Fluorescence detection; ELISA, Enzy-melinked immunosorbent assay; CLIA, Chemiluminescence immunoassay; Simoa, single molecule array; Cut off: pg/mL.

### 3.2 Quality assessment of evidence

**Figure 2:**
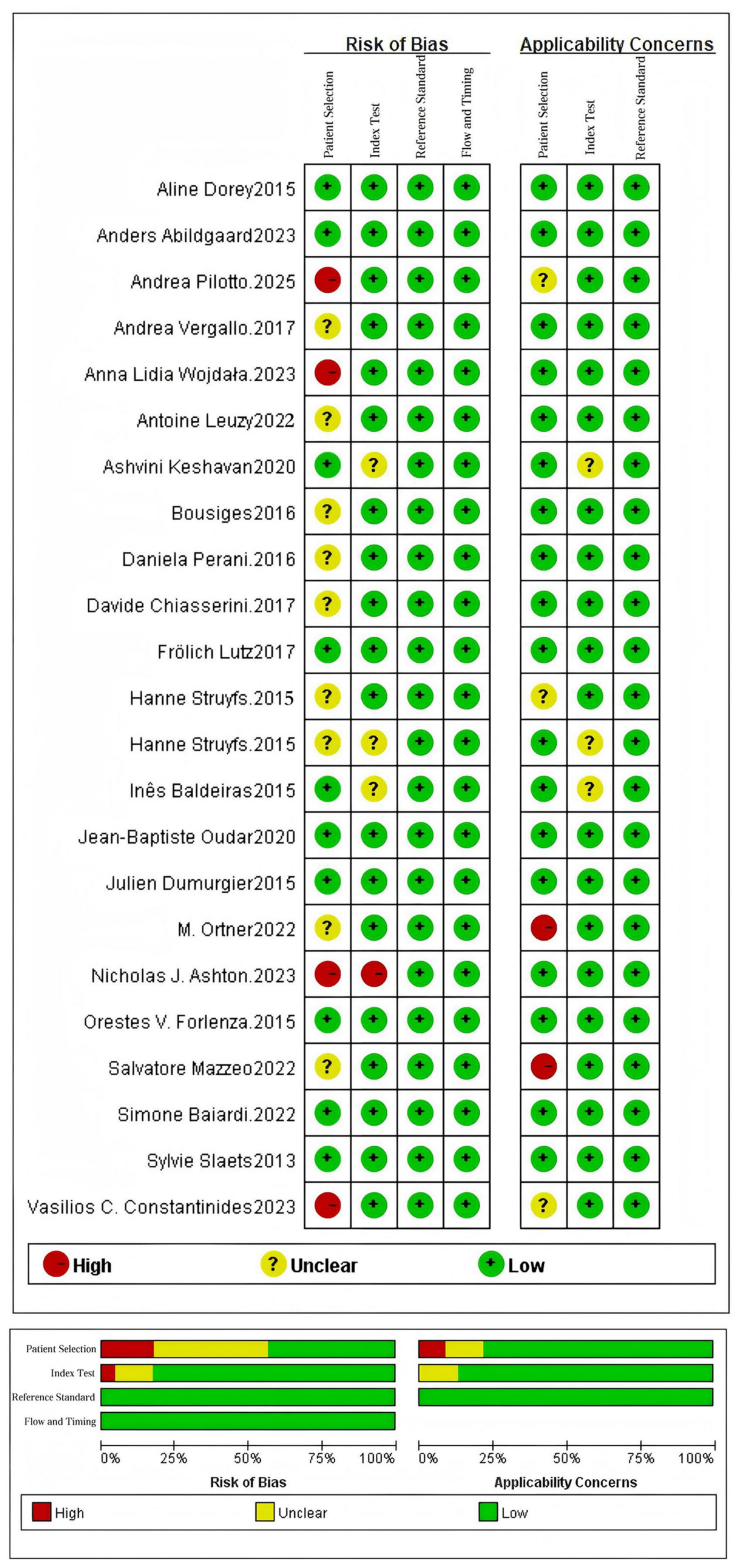
Quality assessment diagram and risk of bias summary.

The methodological quality and risk of bias of the 23 included studies were systematically evaluated using the QUADAS-2 tool across four critical domains: patient selection, index test, reference standard, and flow and timing. The detailed assessment results are presented in Figure 2.

### 3.3 Meta-analytic evidence synthesis outcomes

Comprehensive meta-analytic integration of diagnostic accuracy parameters for the eight core CSF biomarkers was performed using R software, including pooled estimates of sensitivity, specificity, LR+, LR−, DOR, and AUC. The complete statistical synthesis results are presented in Table 2.

**Table 2:** Diagnostic performance analysis of eight core CSF biomarkers.

Biomaker	Study (N)	Case sample size		Sensitivity	Specificity	Positive likelihood ratio	Negative likelihood ratio	Diagnostic odds ratio	AUC
		AD	non-AD						
Aβ42	15	1,312	1,303	0.84(0.79–0.88)	0.65(0.57–0.73)	2.44(1.93–3.10)	0.25 (0.19–0.32)	9.90 (6.74–14.28)	0.85 (0.81–0.90)
t-tau	14	1,232	1,228	0.76 (0.64–0.85)	0.79 (0.75–0.83)	3.70 (2.73–4.81)	0.31 (0.17–0.48)	13.16 (5.86–26.12)	0.82 (0.77–0.87)
p-tau181	12	1,093	1,158	0.85 (0.81–0.89)	0.77 (0.71–0.82)	3.76 (2.74–5.05)	0.19 (0.13–0.27)	20.83 (10.76–36.94)	0.90 (0.87–0.93)
p-tau217	3	408	232	0.95 (0.92–0.97)	0.94 (0.88–0.98)	20.13 (6.54–54.81)	0.06 (0.03–0.10)	395.28 (92.17–1305.79)	0.99 (0.97–1.00)
p-tau231	3	281	201	0.91 (0.86–0.96)	0.92 (0.87–0.96)	12.24 (6.08–23.64)	0.10 (0.04–0.17)	152.61 (44.26–434.17)	0.97 (0.94–1.00)
Aβ42/Aβ40	12	1,203	1,305	0.84 (0.78–0.90)	0.77 (0.70–0.84)	3.83 (2.58–5.78)	0.20 (0.11–0.32)	20.52 (8.81–43.45)	0.87 (0.83–0.92)
Aβ42/t-tau	8	714	575	0.88 (0.81–0.94)	0.80 (0.73–0.86)	4.61 (2.91–7.09)	0.15 (0.06–0.28)	37.58 (10.98–104.49)	0.90 (0.84–0.96)
Aβ42/p-tau181	8	646	509	0.90 (0.86–0.94)	0.80 (0.73–0.87)	4.76 (3.00–7.52)	0.13 (0.06–0.20)	41.62 (15.88–96.73)	0.93 (0.90–0.96)

#### 3.3.1 Sensitivity analysis: comparative diagnostic sensitivity between individual biomarkers and biomarker ratios

**Figure 3:**
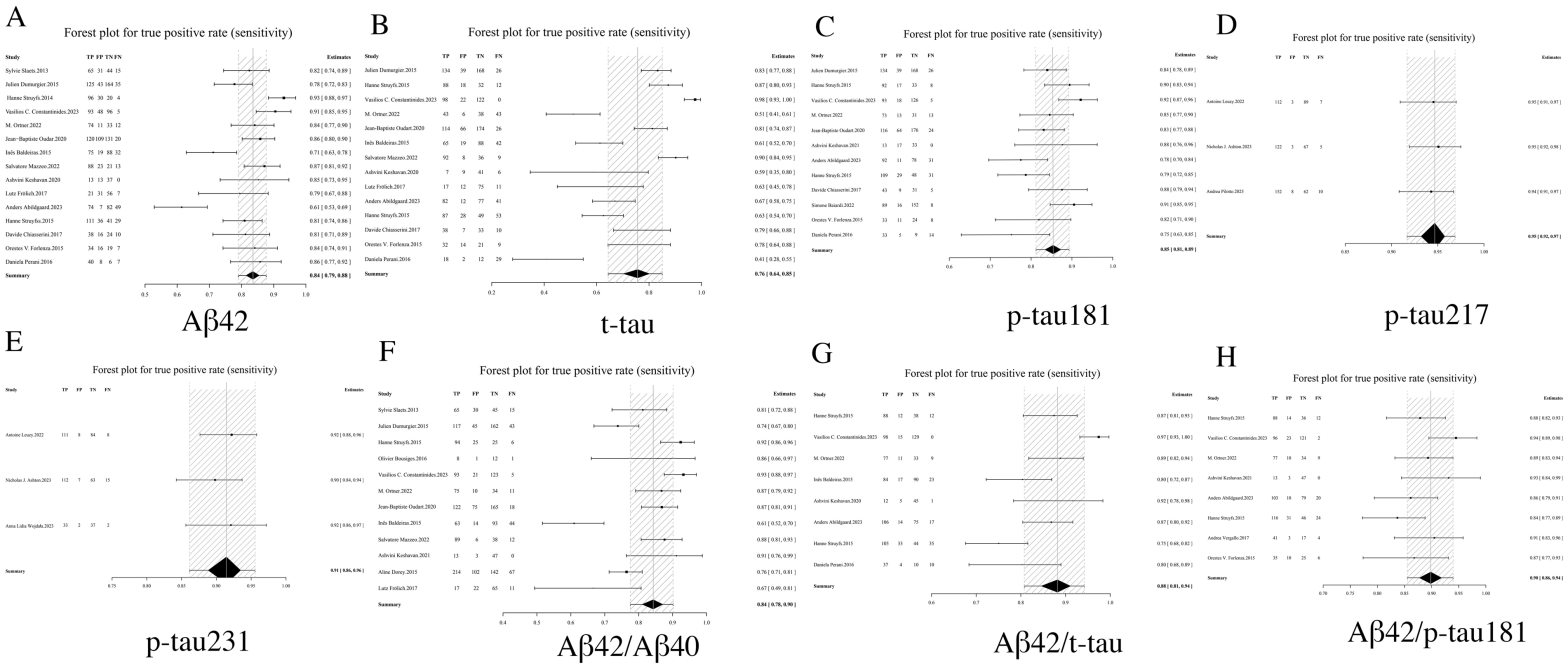
Forest plot of sensitivity for core CSF biomarkers. **(A)** Aβ42, **(B)** t-tau, **(C)** p-tau181, **(D)** p-tau217, **(E)** p-tau231, **(F)** Aβ42/Aβ40, **(G)** Aβ42/t-tau, **(H)** Aβ42/p-tau181.

In AD diagnosis, sensitivity quantifies a biomarker’s ability to correctly identify true AD cases. Among individual biomarkers, p-tau217 demonstrated the highest sensitivity (0.95, 95% CI: 0.92–0.97), yielding only a 5% false-negative rate. Comparative analysis revealed p-tau231 (0.91, 95% CI: 0.86–0.96) and p-tau181 (0.85, 95% CI: 0.81–0.89) as subsequent performers, while t-tau showed the lowest sensitivity (0.76, 95% CI: 0.64–0.85). Notably, biomarker ratios - particularly Aβ42/p-tau181 (0.90, 95% CI: 0.86–0.94) and Aβ42/t-tau (0.88, 95% CI: 0.81–0.94) - consistently outperformed standalone Aβ42 measurement (0.84, 95% CI: 0.79–0.88), indicating the diagnostic advantage of ratio-based approaches. In summary, both p-tau217 and p-tau231 demonstrated superior sensitivity as individual biomarkers, while the Aβ42/p-tau181 ratio exhibited optimal diagnostic performance among biomarker combinations. These findings support their clinical utility as frontline screening tools for AD (see Table 2 and Figure 3 for complete results of Sensitivity).

#### 3.3.2 Specificity analysis: diagnostic discriminatory power of distinct biomarkers

**Figure 4:**
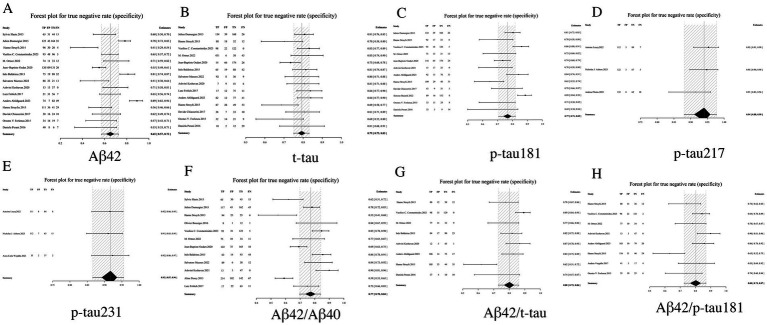
Forest plot of specificity for core CSF biomarkers. **(A)** Aβ42, **(B)** t-tau, **(C)** p-tau181, **(D)** p-tau217, **(E)** p-tau231, **(F)** Aβ42/Aβ40, **(G)** Aβ42/t-tau, **(H)** Aβ42/p-tau181.

Specificity, a critical metric in diagnostic test evaluation, quantifies the ability of biomarkers to accurately exclude non-Alzheimer’s dementia cases. Our comprehensive analysis demonstrated that among individual CSF biomarkers, p-tau 217 (0.94, 95% CI: 0.88–0.98) and p-tau231 (0.92, 95% CI: 0.87–0.96) exhibited the highest specificity profiles, corresponding to clinically favorable false-positive rates of only 6–8%. In contrast, Aβ42 showed substantially lower specificity (0.65, 95% CI: 0.57–0.73), indicating a 39% probability of misclassification. Notably, biomarker ratios - particularly the Aβ42/p-tau181 ratio (0.80, 95% CI: 0.73–0.87) and Aβ42/t-tau ratio (0.80, 95% CI: 0.73–0.86)—demonstrated statistically superior specificity compared to Aβ42 alone, though remained slightly less specific than individual phosphorylated tau variants. Intermediate specificity was observed for standalone p-tau181 (0.77, 95% CI: 0.71–0.82) and t-tau (0.79, 95% CI: 0.75–0.83) measurements. Importantly, the combinatorial approach of biomarker ratios significantly enhanced diagnostic specificity beyond what could be achieved with single biomarkers. These findings collectively position p-tau217 and p-tau231 as optimal confirmatory biomarkers, while supporting the complementary role of ratio-based strategies in mitigating the diagnostic limitations of Aβ42 (see Table 2 and Figure 4 for complete results of Specificity).

#### 3.3.3 LR+ analysis: quantitative assessment of diagnostic certainty

**Figure 5:**
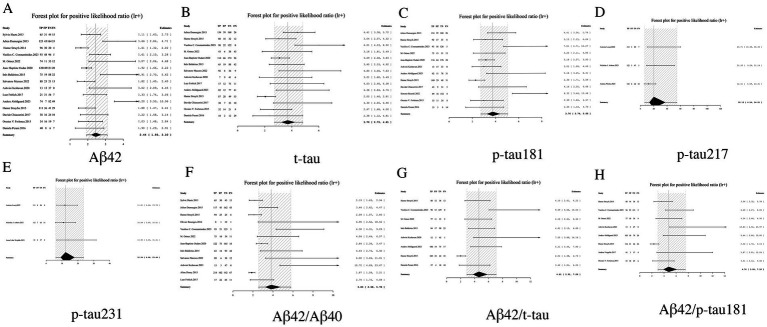
Forest plot of LR+ for core CSF biomarkers. **(A)** Aβ42, **(B)** t-tau, **(C)** p-tau181, **(D)** p-tau217, **(E)** p-tau231, **(F)** Aβ42/Aβ40, **(G)** Aβ42/t-tau, **(H)** Aβ42/p-tau181.

LR+, a key metric in diagnostic test evaluation, quantitatively assesses the probability of confirming AD when biomarker results are positive. Our analysis demonstrated that among individual biomarkers, p-tau217 exhibited an exceptionally high LR+ of 20.13 (95% CI: 6.54–54.81), indicating that a positive result provides near-definitive diagnostic confirmation, significantly outperforming other markers including p-tau231 (12.24, 95% CI: 6.08–23.64) and p-tau181 (3.76, 95% CI: 2.74–5.05). In contrast, Aβ42 showed limited diagnostic utility with the lowest LR + (2.44, 95% CI: 1.93–3.10). While biomarker ratios including Aβ42/p-tau181 (4.76, 95% CI: 3.00–7.52) and Aβ42/t-tau (4.61, 95% CI: 2.91–7.09) demonstrated significantly improved performance over Aβ42 alone, they remained inferior to phosphorylated tau isoforms. These findings establish p-tau217 and p-tau231 as optimal standalone biomarkers for AD confirmation, while supporting the complementary role of ratio-based approaches in enhancing Aβ42’s diagnostic value. From a laboratory medicine perspective, the markedly elevated LR + values (>10) particularly qualify p-tau217 and p-tau231 as excellent candidates for confirmatory testing, capable of substantially reducing diagnostic uncertainty in clinical practice (see Table 2 and Figure 5 for complete results of LR+).

#### 3.3.4 LR− analysis: systematic evaluation of disease exclusion capacity

**Figure 6:**
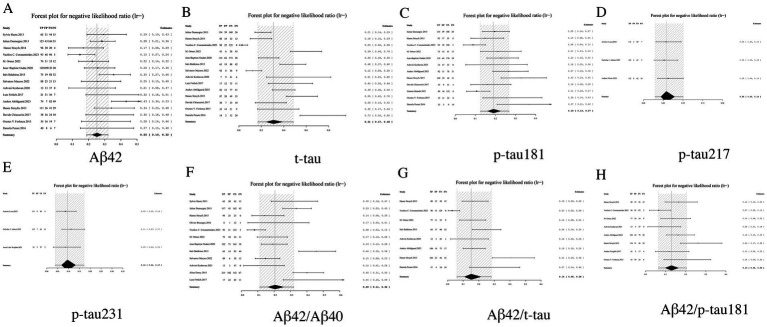
Forest plot of LR− for core CSF biomarkers. **(A)** Aβ42, **(B)** t-tau, **(C)** p-tau181, **(D)** p-tau217, **(E)** p-tau231, **(F)** Aβ42/Aβ40, **(G)** Aβ42/t-tau, **(H)** Aβ42/p-tau181.

LR− serves as a critical metric for assessing the reliability of excluding AD when biomarker results are negative, with lower values indicating superior diagnostic performance. Among individual biomarkers, p-tau217 demonstrated exceptional exclusion capacity with an LR− of 0.06 (95% CI: 0.03–0.10), suggesting near-complete certainty in ruling out AD when negative. Similarly outstanding performance was observed for p-tau231 (0.10, 95% CI: 0.04–0.17) and p-tau181 (0.19, 95% CI: 0.13–0.27). Biomarker ratios including Aβ42/p-tau181 (0.13, 95% CI: 0.06–0.20) and Aβ42/t-tau (0.15, 95% CI: 0.06–0.28) showed significantly improved exclusion capability compared to Aβ42 alone (0.25, 95% CI: 0.19–0.32), though remained slightly inferior to individual phosphorylated tau isoforms. Notably, t-tau exhibited the least favorable exclusion profile (LR− = 0.31, 95% CI: 0.17–0.48). These findings establish p-tau217 and p-tau231 as optimal biomarkers for AD exclusion, while supporting the complementary role of ratio-based approaches in enhancing the negative predictive value of Aβ42. Based on these results, we strongly recommend prioritizing biomarkers with LR− values <0.1 in clinical screening algorithms to maximize diagnostic certainty (see Table 2 and Figure 6 for complete results of LR−).

#### 3.3.5 DOR analysis: systematic evaluation of comprehensive diagnostic performance

**Figure 7:**
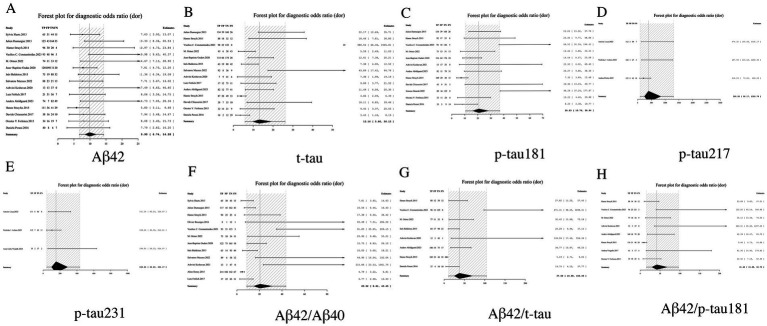
Forest plot of DOR for core CSF biomarkers. **(A)** Aβ42, **(B)** t-tau, **(C)** p-tau181, **(D)** p-tau217, **(E)** p-tau231, **(F)** Aβ42/Aβ40, **(G)** Aβ42/t-tau, **(H)** Aβ42/p-tau181.

DOR analysis demonstrated superior integrated diagnostic performance for p-tau217, with an exceptionally high DOR of 395.28 (95% CI: 92.17–1305.79). The performance hierarchy revealed p-tau231 (DOR = 152.61, 95% CI: 44.26–434.17) and the Aβ42/p-tau181 ratio (DOR = 41.62, 95% CI: 15.88–96.73) as secondary top performers. Notably, all biomarker ratios exhibited significantly enhanced DOR values compared to their corresponding individual biomarkers, exemplified by the Aβ42/t-tau ratio (DOR = 37.58, 95% CI: 10.98–104.49) demonstrating marked improvement over Aβ42 alone (DOR = 9.90, 95% CI: 6.74–14.28). These findings robustly validate the diagnostic superiority of ratio-based approaches. From a clinical implementation perspective, biomarkers with DOR > 100 (p-tau217 and p-tau231) qualify as potential gold-standard indicators for AD diagnosis, while ratio markers with DOR > 30 may serve as valuable alternatives in clinical algorithms (see Table 2 and Figure 7 for complete results of DOR).

#### 3.3.6 AUC analysis: comprehensive evaluation of overall diagnostic accuracy

**Figure 8:**
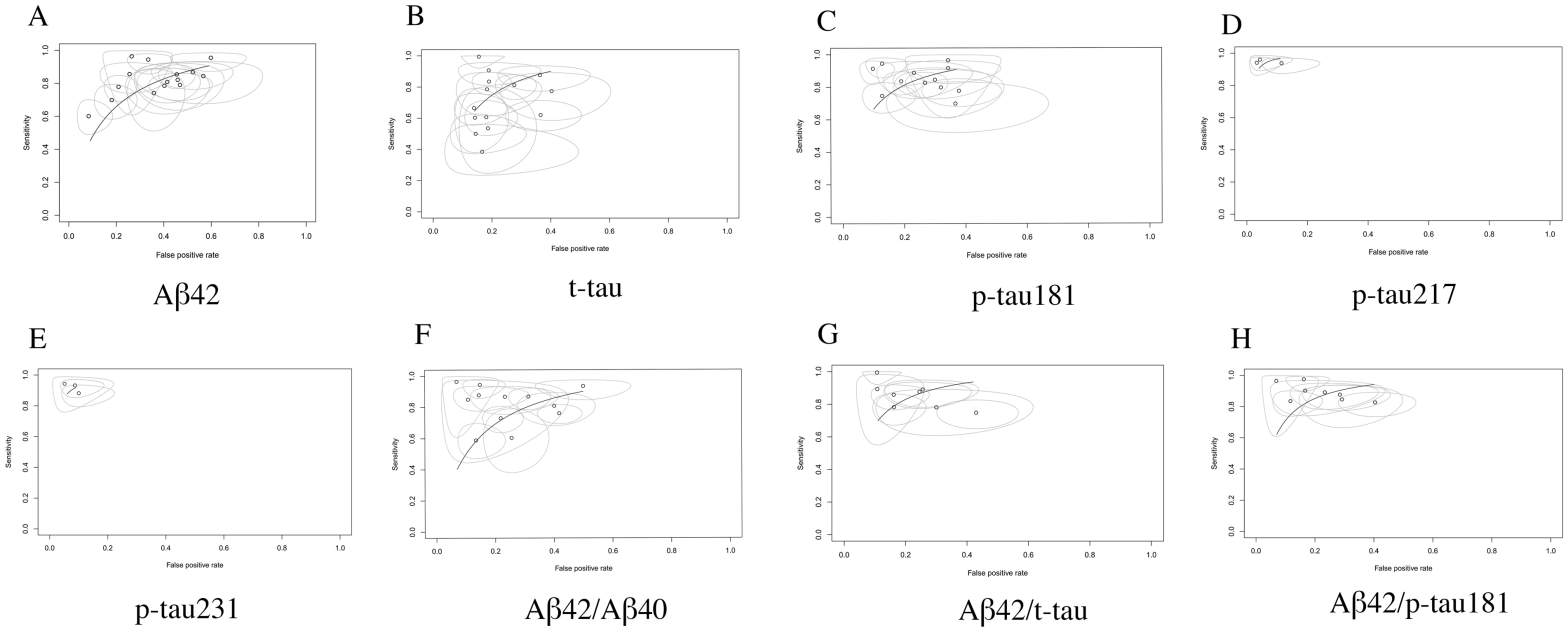
SROC curve analysis of core CSF biomarkers. **(A)** Aβ42, **(B)** t-tau, **(C)** p-tau181, **(D)** p-tau217, **(E)** p-tau231, **(F)** Aβ42/Aβ40, **(G)** Aβ42/t-tau, **(H)** Aβ42/p-tau181.

AUC analysis demonstrated superior diagnostic performance among CSF biomarkers for AD detection. Specifically, p-tau217 exhibited near-perfect discriminative capacity with an AUC of 0.99 (95% CI: 0.97–1.00), followed closely by p-tau231 (AUC = 0.97, 95% CI: 0.94–1.00) and the Aβ42/p-tau181 ratio (AUC = 0.93, 95% CI: 0.90–0.96). Notably, biomarker combinations consistently achieved higher AUC values compared to individual biomarkers, highlighting the diagnostic advantage of ratio-based approaches. According to established diagnostic accuracy benchmarks: Biomarkers with AUC values exceeding 0.90 (p-tau217, p-tau231, and Aβ42/p-tau181) are classified as having excellent diagnostic utility; Markers with AUC values between 0.80–0.90 (p-tau181 and Aβ42/t-tau ratio) demonstrate good but relatively inferior performance (15) (see Table 2; performance gradients illustrated in Figure 8).

#### 3.3.7 Heterogeneity analysis

##### 3.3.7.1 Threshold effect heterogeneity analysis in diagnostic accuracy studies

The Spearman’s rank correlation analysis demonstrated that among the eight CSF biomarkers evaluated, only p-tau231 exhibited significant threshold effect (*p* < 0.05). In contrast, the remaining seven core CSF biomarkers showed no statistically significant threshold effect (all *p*-values >0.05), indicating the absence of threshold-induced heterogeneity in these markers. This finding suggests that for most biomarkers examined (87.5%), diagnostic accuracy remains consistent across different cutoff values, with p-tau231 being the sole exception requiring careful threshold optimization in clinical applications. Detailed correlation coefficients and corresponding p-values are presented in Table 3.

**Table 3:** Heterogeneity analysist results induced by threshold effects.

Biomaker	Spearman correlation coefficient	*p*-value
Aβ42	0.39	0.16
t-tau	0.41	0.14
p-tau181	−0.20	0.53
p-tau217	−0.50	0.67
p-tau231	−1.00	0.00
Aβ42/Aβ40	−0.25	0.43
Aβ42/t-tau	−0.69	0.06
Aβ42/p-tau181	−0.55	0.16

##### 3.3.7.2 Non-threshold effect heterogeneity analysis in diagnostic accuracy studies

The Cochran’s Q test for heterogeneity across the eight core CSF biomarkers revealed significant non-threshold effect induced variability in diagnostic odds ratios (DORs). While Aβ42, p-tau217, and p-tau231 demonstrated relative homogeneity (*p* ≥ 0.01), all remaining biomarkers showed statistically significant heterogeneity (*p* < 0.01). Quantitative assessment using I^2^ statistics further confirmed this pattern: only Aβ42, p-tau217, and p-tau231 exhibited moderate heterogeneity (I^2^ < 50%) across effect sizes, whereas other biomarkers displayed substantial variability (I^2^ > 50%) in all diagnostic parameters including sensitivity, specificity, positive/negative likelihood ratios, and DORs. Therefore, the results of this study were analyzed using a random effects model for Bayesian meta-analysis.

#### 3.3.8 Publication bias

**Figure 9:**
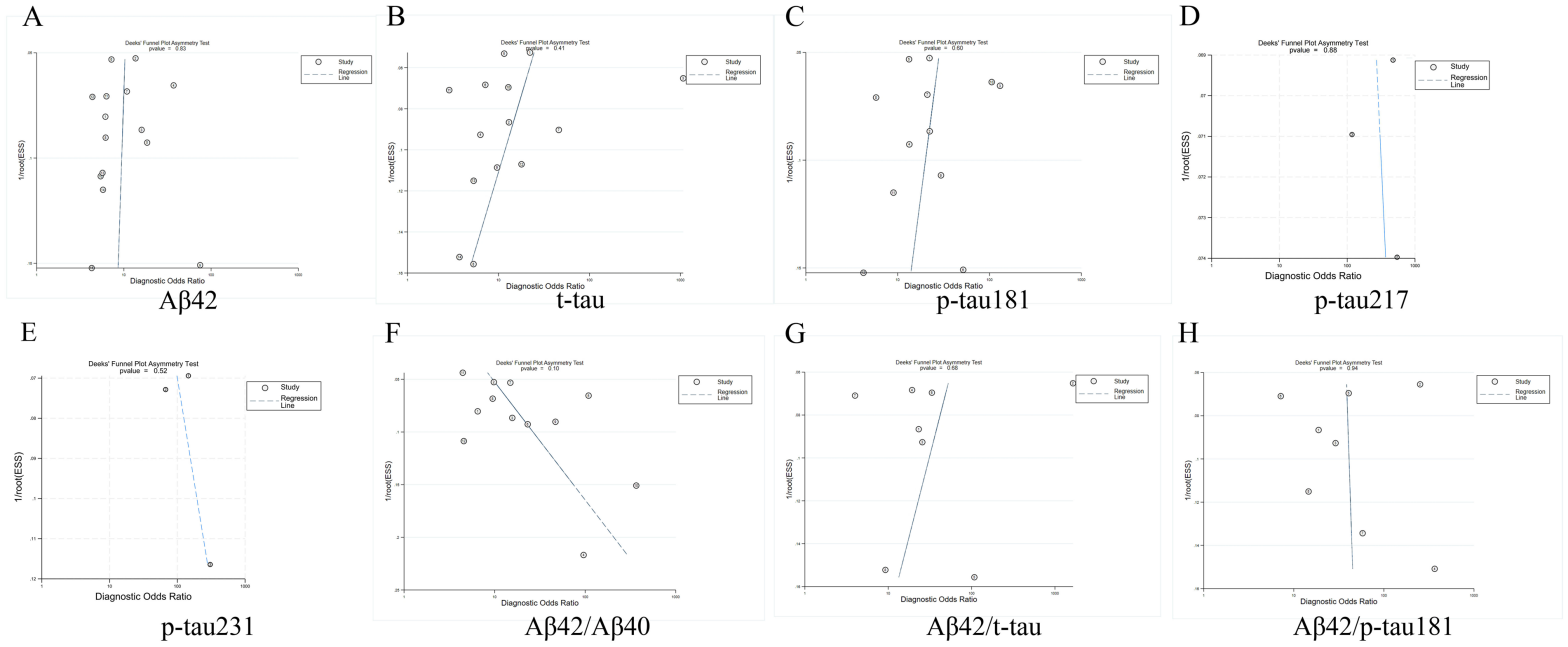
Deeks funnel chart. **(A)** Aβ42, **(B)** t-tau, **(C)** p-tau181, **(D)** p-tau217, **(E)** p-tau231, **(F)** Aβ42/Aβ40, **(G)** Aβ42/t-tau, **(H)** Aβ42/p-tau181.

Deeks’ funnel plot analysis revealed no significant publication bias among the eight evaluated core CSF biomarkers (all *p*-values >0.05), indicating balanced representation of studies in our meta-analysis. The results are shown in Figure 9.

## 4 Discussion

In the current research, CSF biomarkers represent the longest-studied and most comprehensively analyzed indicators for AD, effectively reflecting the pathological changes in affected patients. The Aβ protein indicates Aβ deposition, while p-tau181 signifies the excessive phosphorylation of tau protein and the formation of neurofibrillary tangles. Currently, both Aβ and tau pathological markers, recognized as core CSF indicators in AD (16). As research progresses, the ratios of CSF (Hybrid ratios) demonstrate superior diagnostic performance compared to individual markers, providing unique diagnostic value in differentiating AD patients from those without the condition. Specifically, the ratio of Aβ subtypes (e.g., Aβ42/Aβ40) is believed to mitigate the influence of individual variability and certain objective analytical factors, outperforming individual Aβ polypeptide subtypes (9, 10), Furthermore, the Aβ42 to tau protein ratio exhibits enhanced diagnostic efficacy over single biomarkers in distinguishing AD patients from non-affected individuals. However, there remains a lack of evidence to ascertain which ratio, between Aβ42/Aβ40 and Aβ42 to tau protein, possesses higher diagnostic accuracy (17).

To further optimize diagnostic strategies for AD in clinical practice, this study comprehensively evaluated the diagnostic performance of various core CSF biomarkers in distinguishing patients with AD from those without it, utilizing the Bayesian meta-analysis method. The results indicated that the overall diagnostic performance of the CSF biomarker ratios Aβ42/Aβ40, Aβ42/t-tau, and Aβ42/p-tau181 surpassed that of the individual markers Aβ42, t-tau, and p-tau181, yet was inferior to that of the individual markers p-tau217 and p-tau231. Notably, compared to p-tau181, the Aβ42/Aβ40 ratio demonstrates superior diagnostic performance. Furthermore, the diagnostic performance of Aβ42/t-tau is very similar to that of p-tau181. Recent studies have highlighted that p-tau217 and p-tau231, which are related to Phosphorylated tau protein, exhibit strong correlations with the pathologies of Aβ and tau, while also demonstrating excellent diagnostic performance (18, 19). The results of the meta-analysis may further reveal a strong correlation between phosphorylated tau protein, represented by p-tau181, and Alzheimer’s disease-related Aβ and tau pathology. This indicates the unique advantage of p-tau181 in distinguishing Alzheimer’s disease from other conditions. In the comparison of different types of CSF biomarkers and their ratios, p-tau217, p-tau231, and Aβ42/p-tau181 demonstrated the best diagnostic performance, with high values for key indicators such as sensitivity, specificity, and AUC.

The results of this study provide a significant foundation for the application of core CSF biomarkers in diagnosing AD. The p-tau217 biomarker demonstrates high diagnostic performance, suggesting a strong correlation between p-tau217 and the pathology of amyloid-beta (Aβ) and tau in AD (20). In the future, more comprehensive diagnostic tests should be conducted for various threonine sites, such as p-tau217, p-tau231, and p-tau205. Additionally, the ratios of core CSF biomarkers, particularly the ratio of Aβ42 protein to tau protein (e.g., Aβ42/p-tau181), have demonstrated superior diagnostic performance compared to the Aβ42/Aβ40 ratio. This maximizes the diagnostic accuracy of core CSF biomarkers in differentiating AD from non-AD. This suggests that the Aβ42 to p-tau181 ratio will increasingly play a crucial role in distinguishing AD from other conditions with similar clinical manifestations. However, CSF testing necessitates lumbar puncture, a procedure that is complex and often results in low patient compliance (21). Therefore, it is essential to further optimize detection methods to enhance their convenience and acceptability. Additionally, ongoing research that combines other non-invasive or minimally invasive markers, such as blood markers, is expected to provide a more comprehensive solution for the early diagnosis of AD (22).

Although this study provides valuable insights, it is not without limitations. Firstly, the primary detection method for core CSF biomarkers employed in the included studies was ELISA, which may have constrained the generalizability of the findings (23). Second, the included studies contain retrospective studies, which may bring about certain heterogeneity (24). Third, the included studies lacked pathological confirmation, potentially leading to false-positive or false-negative results. The last point highlights the heterogeneity present in the included research sections. Additionally, beyond the eight core CSF biomarkers mentioned, other biomarkers such as p-tau205 should also be considered. However, due to the limited number of studies available, no research meeting the necessary criteria for in-depth analysis has been identified. Future studies should integrate clinical practice, include a broader range of core CSF biomarkers, verify the diagnostic efficacy of these biomarkers across diverse populations, and investigate other potential biomarkers.

## 5 Conclusion

This study employs a Bayesian meta-analysis approach to confirm the significance of core CSF biomarkers in the diagnosis of AD, particularly the single biomarker p-tau217 and the biomarker ratio Aβ42/p-tau181. The high AUC values and sensitivity of these biomarkers indicate their substantial potential for early identification of AD patients, providing valuable reference tools for clinicians. Future research will further expand the sample size, delve into the mechanisms of action of core CSF biomarkers in AD, and optimize diagnostic methods to enhance the diagnostic accuracy and clinical applicability of AD.
